# The general surgeon’s perspective of rectus diastasis. A systematic review of treatment options

**DOI:** 10.1007/s00464-017-5607-9

**Published:** 2017-06-08

**Authors:** Elwin H. H. Mommers, Jeroen E. H. Ponten, Aminah K. Al Omar, Tammo S. de Vries Reilingh, Nicole D. Bouvy, Simon W. Nienhuijs

**Affiliations:** 1 Department of Surgery, Maastricht University Medical Center, Maastricht, The Netherlands; 2 Department of Surgery, Catharina Hospital, Eindhoven, The Netherlands; 3 Department of Surgery, Elkerliek Hospital, Helmond, The Netherlands

**Keywords:** Diastasis of the rectus abdominis muscles (DRAM), Diastasis repair, Surgical treatment, Physiotherapy

## Abstract

**Background:**

Diastasis of the rectus abdominis muscles (DRAM) is characterised by thinning and widening of the linea alba, combined with laxity of the ventral abdominal musculature. This causes the midline to “bulge” when intra-abdominal pressure is increased. Plastic surgery treatment for DRAM has been thoroughly evaluated, though general surgical treatments and the efficacy of physiotherapy remain elusive. The aim of this systematic literature review is to evaluate both general surgical and physiotherapeutic treatment options for restoring DRAM in terms of postoperative complications, patient satisfaction, and recurrence rates.

**Method:**

MEDLINE^®^, Embase, PubMed, PubMed Central^®^, The cochrane central registry of controlled trials (CENTRAL), Google Scholar, and the Physiotherapy Evidence Database (PEDro) were searched using the following terms: ‘rectus diastasis’, ‘diastasis recti’, ‘midline’, and ‘abdominal wall’. All clinical studies concerning general surgical or physiotherapeutic treatment of DRAM were eligible for inclusion.

**Result:**

Twenty articles describing 1.691 patients (1.591 surgery/100 physiotherapy) were included. Surgical interventions were classified as plication techniques (313 patients; 254 open/59 laparoscopic), modified hernia repair techniques (68 patients, all open), and combined hernia & DRAM techniques (1.210 patients; 1.149 open/40 hybrid). The overall methodological quality was low. Plication techniques with interrupted sutures and mesh reinforcement were applied most frequently for DRAM repair. Open repairs were performed in 85% of patients. There was no difference in postoperative complications or recurrence rate after laparoscopic or open procedures, or between plication and modified hernia repair techniques. Physiotherapy programmes were unable to reduce IRD in a relaxed state. Though reduction of IRD during muscle contraction was described.

**Conclusion:**

Both plication-based methods and hernia repair methods are used for DRAM repair. Based on the current literature, no clear distinction in recurrence rate, postoperative complications, or patient reported outcomes can be made. Complete resolution of DRAM, measured in a relaxed state, following a physiotherapy training programme is not described in current literature. Physiotherapy can achieve a limited reduction in IRD during muscle contraction, though the impact of this finding on patient satisfaction, cosmesis, or function outcome is unclear.

Diastasis of the rectus abdominis muscles (DRAM) is characterised by a protruding midline following an increase in intra-abdominal pressure. The condition is characterised by a gradual thinning and widening of the linea alba, combined with a general laxity of the ventral abdominal wall muscles [1]. DRAM is frequently misclassified as a primary ventral hernia, though the musculofascial continuity of the midline and subsequent absence of a true hernia sac is what sets DRAM apart from a ventral hernia. DRAM is defined according to the Beer classification as an inter-rectus distance (IRD) of 22 mm, three centimetres above the umbilicus measured in a relaxed state [2]. DRAM occurs most frequently during pregnancy and regresses spontaneously after childbirth in most women. However, 12 months postpartum, 33% of women still experience DRAM [3].

Patients with DRAM can experience similar complaints as patients with ventral hernias, such as lower back pain, functional, and cosmetic impairment, although DRAM does not pose any threat of strangulation [4–6]. DRAM repair is challenging for most general surgeons since guidelines on indication and methods for repair do not exist. The similarity to primary ventral hernias causes frequent misclassification of the disease, and potential mistreatment of DRAM. In recent years, the overall complexity of evidence concerning DRAM treatment has increased. This is due to the development and implementation of several new reconstructive techniques, combined with heterogenous outcome measurements, heterogenous definitions for DRAM, and the lack of high quality data.

DRAM is mostly treated conservatively, with or without the help of a physiotherapist. If conservative therapy is preferred, patients can be referred to a physiotherapist for training programmes that specifically target DRAM, with the aim of reducing IRD and improvement of quality-of-life (QoL). Benjamin et al. evaluated the efficacy of these training programmes in 2014, though due to the low quality of the included studies, no conclusions could be drawn [7].

In case of severe functional or cosmetic impairment, the patient can be referred to a plastic or general surgeon. Patients that suffer from excess skin or want to tailor their waistline simultaneously with DRAM repair should be referred to a surgeon in the field of plastic and reconstructive surgery for an abdominoplasty. Publications describing DRAM repair in combination with abdominoplasty, liposuction, or other strictly plastic surgical techniques are numerous. A recent review of Akram et al. in 2014 concerning abdominoplasty repairs in combination with plication of the linea alba concluded that most evidence is of low quality and RCTs are required to gain more insight in the short- and long-term effects of these combined procedures [8].

Patients with the sole diagnosis of DRAM are frequently referred to the general surgeon. If surgical treatment is considered, several techniques ranging from laparoscopic, endoscopic, hybrid, and open repairs are available. Currently, there is no consensus on the preferred surgical management of DRAM. In contrast to evidence from the reconstructive field, a thorough literature review comparing different surgical techniques for DRAM is noticeably absent in general surgery. The aim of this systematic literature review is to provide insight in the general surgical treatment options for DRAM in terms of postoperative complications, patient satisfaction, and recurrence rates, and to evaluate if physiotherapy is an alternative for surgical intervention.

## Methods

This review was registered on PROSPERO [No.: CRD42016048176], and conducted according to the PRISMA statement [9]. Before the start of the review process, the review protocol was evaluated and approved by an independent, external expert in the field of ventral hernia repair.

### Search strategy

A structured literature search of MEDLINE^®^, Embase, PubMed, PubMed Central^®^ (PMC), The cochrane central registry of controlled trials (CENTRAL), Google Scholar, and the Physiotherapy Evidence Database (PEDro) was performed by two independent reviewers (E.M. and A.A.O.) using the following terms:

‘Diastasis recti’ OR ‘rectus diastasis’ OR ‘diastasis of the rectus abdominis’ OR ‘diastasis of the recti’ OR ‘abdominal diastasis’ OR ‘abdominal separation’ OR ‘diastasis recti abdominis’ OR ‘separation of the recti’ OR ‘separation of the rectus abdominis’ OR ‘divarication of the recti’ OR ‘divarication the rectus abdominis’ (See Table 1 for PubMed search algorithm).

**Table 1:** Search algorithm for PubMed search

((((((((((((diastasis[All Fields] AND recti[All Fields]) OR (rectus[All Fields] AND diastasis[All Fields])) OR (diastasis[All Fields] AND (“rectus abdominis”[MeSH Terms] OR (“rectus”[All Fields] AND “abdominis”[All Fields]) OR “rectus abdominis”[All Fields]))) OR (diastasis[All Fields] AND recti[All Fields])) OR ((“abdomen”[MeSH Terms] OR “abdomen”[All Fields] OR “abdominal”[All Fields]) AND diastasis[All Fields])) OR ((“abdomen”[MeSH Terms] OR “abdomen”[All Fields] OR “abdominal”[All Fields]) AND (“divorce”[MeSH Terms] OR “divorce”[All Fields] OR “separation”[All Fields]))) OR (diastasis[All Fields] AND recti[All Fields] AND abdominis[All Fields])) OR ((“divorce”[MeSH Terms] OR “divorce”[All Fields] OR “separation”[All Fields]) AND recti[All Fields])) OR ((“divorce”[MeSH Terms] OR “divorce”[All Fields] OR “separation”[All Fields]) AND (“rectus abdominis”[MeSH Terms] OR (“rectus”[All Fields] AND “abdominis”[All Fields]) OR “rectus abdominis”[All Fields]))) OR (diastasis[All Fields] AND rectus[All Fields] AND abdominus[All Fields])) OR (divarication[All Fields] AND recti[All Fields])) OR (divarication[All Fields] AND rectus[All Fields] AND abdominus[All Fields])) OR (divarication[All Fields] AND (“rectus abdominis”[MeSH Terms] OR (“rectus”[All Fields] AND “abdominis”[All Fields]) OR “rectus abdominis”[All Fields])))	

Search algorithm for PudMed database search, performed on 8th of September 2016

The last search was performed on 8 September 2016. The search was performed using validated methods of the Cochrane collaboration [10]. Both medical subject heading (MeSH) terms and free-text terms were used to construct the search algorithm. To create a sensitive algorithm, only one domain of search terms (DRAM population) was used. In addition to the above-mentioned database searches, all reference lists of included studies were cross-referenced to retrieve additional articles eligible for inclusion. In case of disagreement between the reviewers regarding the eligibility for inclusion of an article, the study quality, or the data abstraction, a third reviewer (NB) was consulted for arbitration.

### Outcome definition and study selection criteria

The primary outcome for surgical studies was recurrence rate, secondary outcomes were complication rate within 30 days, and patient satisfaction.

The primary outcome for physiotherapy studies was the effect of the treatment on IRD, secondary outcomes were were patient satisfaction, and recurrence rate.

Physiotherapy studies reporting the effect of a single exercise, performed only once, on IRD were considered as functional anatomy studies; these studies were not included in this systematic review. Physiotherapy studies focussing on rectus diastasis during pregnancy or during the immediate postpartum period (≤24 h after childbirth) are not included in this review since results obtained during this period cannot be translated to a later time point, and therefore, do not provide an answer to the secondary endpoint of this review.

Any study (comparative, non-comparative, randomised, or observational studies) describing the effect of a surgical or physiotherapy intervention for DRAM in at least one patient ≥18 years old and reporting on the primary and/or secondary outcome of this systematic literature review was eligible for inclusion, with the exclusion of plastic surgery interventions such as abdominoplasty or liposuction. No limitations to subjects, type of article, or language were applied, though articles published before 1975 which described techniques that have not been applied or described since 1975 were excluded. Articles reporting on the treatment of DRAM combined with primary ventral midline hernias were included, yet analysed separately.

### Quality assessment

The quality of randomised controlled trials (RCTs) was evaluated using a 14-item modified Jadad score and the Cochrane risk of bias tool [11, 12]. The quality of non-randomised clinical studies was evaluated using the methodological index for non-randomised studies (MINORS) criteria [13]. In case of missing data for the quality assessment tool or data abstraction, the first and corresponding author of the study was contacted via email for completion of the missing data. If the author did not respond, a reminder was sent after two weeks. Studies which scored below three on the MINORS checklist were excluded due to lack of critical data needed for correct interpretation of the results, combined with an unacceptable risk of bias, as at least six of eight domains of the MINORS checklist are either not reported, or partially reported in those publications.

### Data abstraction

Data abstraction was performed in duplicate by two independent reviewers (E.M. and A.A.O.) with a standardised electronic data extraction form including but not limited to the following study variables: title, source, year of publication, study design incl. retrospective or prospective design, demographics of study population, sample size, type of rectus diastasis according to Nahas et al., description of intervention, type of analysis (intention to treat vs per protocol), complications within and after 30 days postoperative, follow-up period, follow-up assessment tool, risk factors for recurrence, and recurrence rate [14]. The Nahas classification defines four different aesthetic types of DRAM: Type A, DRAM secondary to pregnancy; Type B, DRAM and laxity of the lateral and infra-umbilical aponeurosis; Type C, congenital lateral insertion of the rectus abdominis muscles; and Type D, DRAM combined with poor waistline.

## Results

**Fig. 1:**
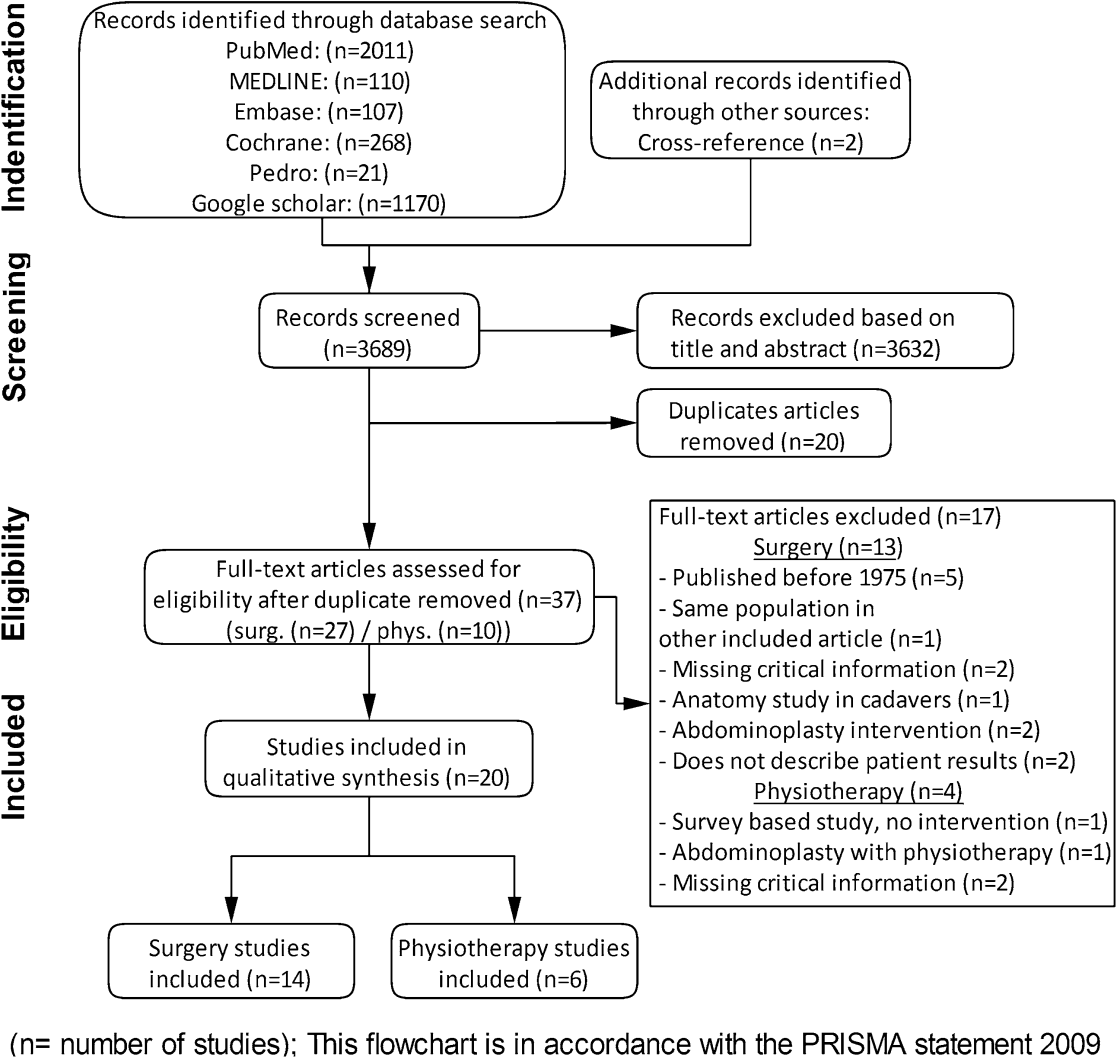
Flow of trials through review. PRISMA flowchart of study selection

After screening 3.689 citations and removal of 20 duplicate manuscripts, 37 articles were selected for full-text review (Fig. 1). Seventeen articles did not meet the inclusion criteria. Twenty studies describing a total of 1.691 patients, 1.591 (94 males, 1497 females) in general surgical techniques and 100 (all female) in physiotherapy training programmes, were included in this systematic review. Fourteen articles described surgical techniques, and six described physiotherapy interventions (Table 1). Five articles were written in Russian, and one in Italian. The included surgical techniques were divided into three categories: (1) plication techniques for DRAM repair, (2) hernia repair techniques modified for DRAM repair, and (3) techniques for DRAM associated with small midline hernias.

## Surgery

### Plication techniques

Six retrospective studies described a surgical technique that included plication of the midline, anterior, or posterior rectus fascia, whilst maintaining the myofascial continuity of the ventral abdominal wall [15–20]. A total of 313 patients were included in this section. Four studies were case series, and two were case reports (Table 2). Quality of the included studies was low–to-moderate with MINORS scores ranging from 4 to 7.

**Table 2:** Eevidence table surgery

Author (year of publication)	Study period	Type of study	Population				Intervention	Primary and secondary outcome	Complications and/or results	Follow-up period (Method)	Recurrence rate %	Study quality
			*N* = …	Age (years)	BMI (Kg/m^2^)	Type of DRAM*						
Plication												
Shirah, B.H. (2016)	2004–2013	Case series	216	40.9	26.4	NR (mean IRD 10 cm)	Open repair with plication of posterior rectus fascia and sublay polypropylene mesh (*n* = 179). Laparoscopic repair with midline plication with continuous suture and intra-abdominal polypropylene mesh (*n* = 37).	Recurrence rate, postop. complications, abdominal wall function, cosmetic outcome	Open: Wound infection (*n* = 11) 6.1% Seroma (*n* = 9) 5% Hematoma (*n* = 5) 2.8% Cosmetic (exc/good 95,6%, unsatis. 4,4%) Laparoscopic: Pain (*n* = 4) 10.8% Cosmetic (exc/good 91,2%, unsatis. 8,1%))	24 months (CT+ clinical examination)	0%	7/16
Sahoo, M.R. (2014)	NR	Case series	3	35–45	NR	A, B	Laparoscopic midline plication with interrupted sutures and intra-abdominal mesh reinforcement.	Recurrence rate, postop. complications	Pain and tightness of abdomen observed (no specification), which decreased during follow-up.	12 months (NR_	0%	5/16
Siddiky, A.H. (2010)	NR	Case report	1	38	NR	B	Laparoscopic midline plication with interrupted mattress sutures without mesh reinforcement.	Recurrence rate, postop. Complications	Postoperative pain and ileus described, delayed discharge after 5 days.	8 weeks (NR)	0%	4/16
Palanivelu, C. (2009)	1998–2007	Case series	18	42	28.2	A, B, D	Laparoscopic midline plication with interrupted venetian blinds sutures and mesh reinforcement.	Recurrence rate, postop. complications	Pain (*n* = 2) 11.1% Pneumonia (*n* = 1) 5.5% Chronic pain(*n* = 2) 11.1%	6–48 months (CT)	0%	7/16
Nahas, F.X. (2004)	NR	Case report	2	38 & 59	NR	C	Open midline plication of the posterior rectus sheath with interrupted sutures, and anchoring of the anterior rectus fascia to the posterior rectus fascia in the midline.	Recurrence rate, postop. complications	Uneventful recovery and satisfactory cosmetic results.	Case 1: 30 months (CT) Case 2: 6 months (CT)	0%	4/16
Deryugina, M.S. (2001)	NR	Case series	73	45.9	NR	NR	Open repair with midline plication of the linea alba using interrupted sutures mesh sublay reinforcement fine-pored woven Lavsan tape.	Recurrence rate	Missing	1–11 years (NR)	4% (*n* = 3)	4/16
Modified hernia repair method												
Angio, L.G. (2007)	NR	Case series	12	43	NR	NR	Open modified Chevrel technique without entering the abdominal cavity. Midline plasty with onlay mesh reinforcement.	Recurrence rate, cosmetic result, postop. complications	Seroma (*n* = 3) 7.0%	24 months (12 months = CT, 18 and 24 months = clinical examination)	0%	8/16
Gireev, G.I. (1983, 1994, 1997)^!^	1980–1989	Case series	56	NR	NR	NR	Open modified Rives–Stoppa repair without mesh reinforcement.	Recurrence rate, work impairment, pain	9 short-term complications, no work impairment 71.4%, moderate impairment 16%, and severe impairment 0%.	24 months (NR)	0%	3/16
Combined (hernia and DRAM)												
Privett, B.J. (2016)	2013-2015	Case series	58	NR	NR	NR (hernia <4 cm)	Open repair of DRAM with small umbilical hernia. Small umbilical incision and preperitoneal placement of self-adhesive mesh.	Recurrence rate, postop. complications	No postop. complications	NR (NR)	1.7% (*n* = 1)	3/16
Köckerling, F. (2016)	2015-2016	Case series	40	53.6	32.6	NR	ELAR plus for DRAM with umbilical or epigastric hernia. Endoscopic-assisted anterior rectus fascia turn over with mesh augmentation resembles a hybrid version of the modified Chevrel technique with only a small umbilical incision.	Postop. complications	Umbilical necrosis (*n* = 1) 2.5% Impaired wound healing (*n* = 1) 2.5% Seroma (*n* = 1) 2.5% Intermittent pain on exertion (*n* = 3) 7.5%	NA	NA	4/16
Bellido L.A. (2015)	2011-2012	Prospective cohort study	21	37.6	27.4	A, B, C, D (hernia ≥2 cm)	DRAM with umbilical or epigastric hernia ≥2 cm. Endoscopic, subcutaneous, midline plication with V-lock suture, and onlay mesh reinforcement.	Recurrence rate, postop. complications, cosmetic appearance, pain (VAS)	Seroma in suprapubic area (*n* = 5) 23% Subcutaneous emphysema (*n* = 2) 9%	20 months (US + clinical examination)	0%	11/16
Matei, O.A. (2014)	2010–2012	Case series	44	60.2	31.2	NR	Open repair with small umbilical hernia with Rives–Stoppa repair combined with sublay mesh placement.	Postop. complications	Minimal umbilical necrosis (*n* = 1)	NR	NA	3/16
Yurasov, A.V. (2014)	2006–2013	Case series	374 degree I: *n* = 174 degree II: *n* = 162 degree III: *n* = 38	NR	NR	NR	Open Rives–Stoppa like repair for DRAM with umbilical hernia with sublay mesh placement continuous suturing of the posterior and anterior rectus fascia. The abdominal cavity is not opened.	Postop. complications	group I: complication rate 2.5% (Hematoma of subcutaneous fat *n* = 1(1.2%) Ileus *n* = 1 (1.2%)) group II: complication rate 5.4%(Wound infection *n* = 1 (1.3%) Hematoma of subcutaneous fat n = 2 (2.7%) Ileus *n* = 1 (1.3%)) group III: complication rate 5% (Hematoma of subcutaneous fat n = 1 (5%))	NR	NA	5/16
Ranney, B. (1990)	NR	Case series	673	NR	NR	A, B (umbilical hernia)	Open midline plication of DRAM with umbilical hernia. Plication of posterior rectus fascia, and continuous suturing of the rectus muscles and anterior rectus fascia.	Recurrence rate, wound dehiscence	Wound dehiscence not observed.	14.8 years (av) 67.28% of patients (clinical examination)	0%	5/16

* Type of DRAM according to Nahas et al.; (Type A (secondary to pregnancy with and a well-defined waistline), Type B (secondary to pregnancy and do not have adequate tension of the lateral and infra-umbilical areas of the myoapneurotic layer), Type C (congenital lateral insertion of the rectus abdominis at the costal margins and association of umbilical or epigastric hernia), Type D (rectus diastasis and poor waistline definition)); *QoL* Quality-of-life, ^!^ articles combined to one reference because they report on the same population, *NR* not reported, *BMI* body mass index, *DRAM* Diastasis Recti abdominis muscle, *Med* median; *Av* average/mean, *Unsatis*. unsatisfactory results, *CT* computed tomography. *US ultrasound*, study quality was assessed using the methodological index for non-randomised studies (MINORS) score, *IRD* inter-recti distance, *AAW* anterior abdominal wall, Chronic pain: pain >6 weeks, *RCT* randomised controlled trials, *PFDI* pelvic floor disability index, *ODI* Oswestry disability index, PSFS = patient-specific functional score, *VAS* visual analogue scale, *NA* not applicable. *Degree of DRAM* according to Askerhanov classification (Degree I: 22–50 mm; Degree II: 51–80 mm; Degree III: >80 MM); BMI and age are reported as average or median depending on the source article

#### Techniques

**Fig. 2:**
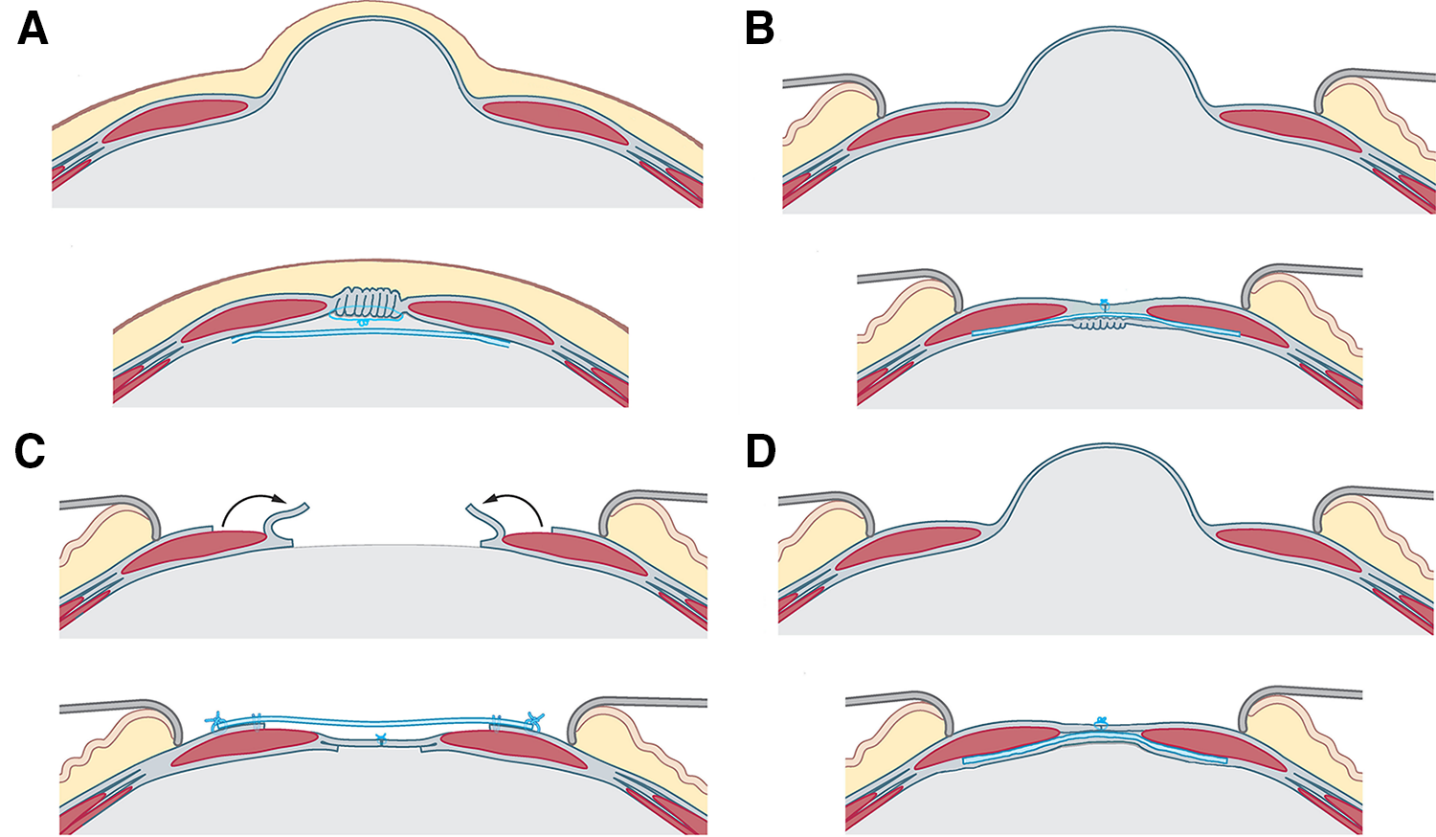
Illustrations of surgical interventions. Four main surgical interventions for treating DRAM. **A** laparoscopic plication of the entire midline with mesh reinforcement, performed in 59 patients; **B** open plication of the posterior rectus fascia, performed in 254 patients; **C** modified Chevrel repair, performed in 52 patients; **D** Rives-Stoppa like repair with or without mesh reinforcement, performed in 948 patients

Four studies used a laparoscopic plication technique (Fig. 2A). All laparoscopic studies used mesh reinforcement, of which three used interrupted sutures, and one used a continuous suture. The study of Palanivelu et al. describes a technique in which they place interrupted sutures which run in and out of the widened linea alba several times, causing the midline to fold like a ‘Venetian blind’ when the sutures are tied [19]. The difference in suture technique did not lead to a difference in recurrence rate, which were 0% in all the laparoscopic repair groups after a follow-up ranging from 6 to 24 months.

Three studies used an open technique, and all plicated a different layer of the ventral abdominal wall (Fig. 2B) [16–18]. The study of Nahas et al. included two female patients with a recurrent DRAM, and is the only study in this section that did not use mesh reinforcement. They describe two type C (congenital lateral implantation of the rectus abdominis muscle) DRAM patients that had undergone plication of the anterior rectus fascia during abdominoplasty surgery and were operated using an open plication technique of the posterior rectus fascia [18]. The anterior rectus fascia was then sutured to the midline, to mimic the anatomic situation. The other two ‘open’ studies described either a plication technique of the anterior rectus fascia or solely the widened linea alba, combined with a sublay mesh.

#### Results of plication techniques

Postoperative complications were reported in five studies. Three studies observed postoperative pain, two of which report an incidence of around 10%. The study of Sahoo et al. clearly shows a surplus of soft tissue clustered at the midline as a direct result of the laparoscopic plication [15]. One of the six studies reported a recurrence after open plication. The original article does not describe recurrences, instead they describe that three patients experienced laxity of the abdominal wall with protrusion, forcing them to wear an abdominal binder. These patients were interpreted as having a recurrent DRAM.

### Modified hernia repair techniques

Two articles used a (modified) hernia repair technique in DRAM patients (Table 2) [21, 22]. The first study from Angio et al. included twelve patients with a midline diastasis and performed a modified Chevrel technique (Fig. 2C) [21]. The modified technique leaves the musculofascial continuity of the ventral abdominal wall intact as the abdominal cavity or the posterior rectus fascia is not opened. Instead, the anterior rectus fascia is incised and overturned to form a wider new posterior rectus fascia. The wident midline is not incised.

The second study of Gireev et al. described a meshless modification of the Rives–Stoppa repair in which the hernia sac is excised and the posterior rectus fascia is sutured using an overlapping plasty. The anterior rectus fascia is then closed [22]. Quality of both studies was 8/16 and 3/16, respectively. The population described by Gireev et al. in the 1997 publication was identical to the population in two prior publications of their group, from 1983 and 1994 [23, 24]. These three publications were combined in one reference and included only once in this review.

#### Results of hernia repair techniques

The study of Angio et al. described seroma formation in 7% of their population. The study of Gireev et al. only reported nine short-term complications, though did not specify these complications in their manuscripts [22]. Both studies had a follow-up of 24 months and observed no recurrences.

### Techniques for combined repair of DRAM and small midline hernia

Six studies, of which five retrospective case series and one prospective cohort study, described surgical treatments for DRAM associated with small midline hernias (Table 2) [6, 25–29]. The total number of patients in this section was 1.210. Quality of the included studies was low-to-moderate as one studies scored 11/16 and the remaining MINORS scores ranged from 3 to 5.

Two studies included both umbilical and epigastric hernias. Four studies included small umbilical hernias. Hernia repair in five out of six studies consisted of suture closure of the hernia sac combined with mesh reinforcement. The oldest study (1990), of Ranney et al., describes a modified Rives–Stoppa repair without mesh reinforcement. Four studies described open techniques, mostly resembling either modifed Chevrel or Rives–Stoppa procedures (Fig. 2C, D  respectively). One described an endoscopic procedure in the anatomical plane between the subcutaneous fat and the anterior rectus fascia, with plication of the midline and onlay mesh reinforcement. One described a hybrid version in the same plane, which resembled a modified Chevrel repair, performed partially endoscopic, with onlay mesh reinforcement.

#### Results of combined repair DRAM and midline hernia

Five of the six studies reported on postoperative complications [6, 25–28]. Two of these studies encountered no postoperative complications, and the remaining three encountered only minor (Clavien-Dindo I–II) postoperative complications. The majority of minor complications were seromas, with an incidence ranging from 2.5%, reported by Köckerling et al. to 23%, reported by Bellido et al. [6, 26].

Follow-up was only reported in two studies and ranged from 20 months to 14.8 years. Only one recurrence was observed during the follow-up in the study of Privett et al. in a patient with a combined DRAM and umbilical hernia [28]. They mentioned that open repair with preperitoneal placement of mesh without approximation of the rectus fascia (resembling a bridged repair) leads to fluctuating cosmetic results, since protrusion of DRAM may still be present after mesh placement.

## Physiotherapy

Six studies evaluated the effect of a physiotherapy intervention on IRD in a total of one hundred postpartum women. Two RCTs, two prospective uncontrolled trials, and two case reports were included in this section (Table 3) [5, 30–34]. All studies focussed on females at different postpartum intervals, ranging from one month to three years. Scientific quality of the included studies was moderate with MINORS scores ranging from six to nine for the non-randomised studies, and a Jadad score of 7–11 for the included RCTs. See Table 4 for results of The Cochrane risk of bias tool.

**Table 3:** Evidence table physiotherapy

Author (year of publication)	Type of study	Population				Intervention	Outcomes	Follow-up period (Method for IRD assessment)	Results	Study quality
		N = … (time postpartum)	Age (years)	BMI (Kg/m^2^)	Type of DRAM*					
Walton, L.M. (2016)	RCT (physiotherapy vs. physiotherapy)	8 (3 months–3 years postpartum)	32.0	27.4	A and B	Traditional group: supine strengthening exercise Experimental group: Core stabilisation including plank, posterior pelvic tilt, kegel, and Russian twist exercises.	IRD PFDI and ODI Disability scores	1.5 months (US & dial caliper, 2 cm above/below umbilicus)	Both groups showed IRD decrease, though traditional therapy showed greater IRD reduction (10.97 to 6.63 cm), PFDI scores did not improve.	JADAD score: 7/13 Cochrane risk of bias tool: (see Table 4)
						Frequency: 3× per week, during 6 weeks (18 sessions)				
Emanuelsson, P. (2016)	RCT (abdominoplasty vs physiotherapy)	30 (1 year postpartum)	44.2	22.8	A & B	Rectus abdominis, Internal/external oblique, and transvers abdominal muscle strengthening exercises	Pain, QoL (SF-36) Muscle strength (Biodex 4)	3 months (NA)	Eighty-seven percent or patients dissatisfied with training results due to continued bulging and functional disability.	JADAD score: 11/13 Cochrane risk of bias tool: (see Table 4)
						Frequency: 3× per week, during 12 weeks (36 sessions)				
Khandale, S.R. (2016)	Prospective uncontrolled trial	30 (NR)	21.7	23.1	A and B	Head lift, pelvic lock, plank, superman, and double leg raise	IRD	2 months (dial caliper & palpation during muscle contraction, 2 cm above/below umbilicus)	IRD decrease above (25.3 mm to 21.9 mm) and below umbilicus (21.9 mm to 19.0 mm) (*p* < 0.0001).	MINORS: 6/16
						Frequency: 5x per week, 30 min per day, during 8 weeks (40 sessions)				
Acharry, N. (2015)	Prospective uncontrolled trial	30 (≤1 month postpartum)	28.8	NR	A and B	Head lift, pelvic tilt, and pelvic clock with bracing	IRD	2 weeks (palpation at umbilicus)	IRD decrease from 3.5 fingerbreadths to 2.5 fingerbreadths (*p* < 0.001).	MINORS 7/16
						Frequency: 2× per day, during 2 weeks (4 sessions)				
Litos, K. (2014)	case report	1(7 weeks postpartum)	32	21.6	A	Core stabilisation exercise and strengthening hip and trunk muscles.	IRD, PSFS & pain scores	4 months (palpation and tape measure during muscle contraction, 4.5 cm above/below umbilicus)	IRD decrease from 11.5 cm to 2.0 cm. Improvement of PSFS score from 4/30 to 30/30.	MINORS: 9/16
						Frequency: 1–2× per week, during 16 weeks (18 sessions)				
Sheppard, S. (1996)	Case report	1 (2 years postpartum)	NR	NR	A or B	Prone kneeling (trans abdominis rehabilitation)	IRD	4 months (tape measure during muscle contraction, location not described)	IRD decrease from 60 mm to 7 mm.	MINORS: 8/16

* Type of DRAM according to Nahas et al.; (Type A (secondary to pregnancy with and a well-defined waistline), Type B (secondary to pregnancy and do not have adequate tension of the lateral and infra-umbilical areas of the myoapneurotic layer), Type C (congenital lateral insertion of the rectus abdominis at the costal margins and association of umbilical or epigastric hernia), Type D (rectus diastasis and poor waistline definition)); *QoL* Quality-of-life, *NR* not reported, *BMI* body mass index, *DRAM* Diastasis Recti abdominis muscle, *Med* median, *Av* average/mean, *CT* computed tomography. *US* ultrasound, *MINORS* methodological index for non-randomised studies, *IRD* inter-recti distance, *AAW* anterior abdominal wall, Chronic pain: pain >6 weeks, *RCT* randomised controlled trials, *PFDI* pelvic floor disability index, *ODI* Oswestry disability index, *PSFS* patient-specific functional score, *VAS* visual analogue scale, *NA* not applicable. *Degree of DRAM* according to Askerhanov classification (Degree I: 22–50 mm; Degree II: 51–80 mm; Degree III: >80 MM). Age and BMI are reported in average or median range depending on the source article

**Table 4:** Cochrane risk of bias tool results

Cochrane risk of bias table of included RCT’s; ‘?’ = unclear risk of bias; ‘-‘=high risk of bias; ‘+’ = low risk of bias

### Training programme

One case study used a single exercise (prone kneeling) to train the patients [33]. Two studies used general (not further specified) strengthening exercises for the abdominal wall, hip, and trunk muscles [5, 32]. The remaining three studies used the head lift exercise, combined with pelvic lock or pelvic tilt exercises [30, 31, 34].

 Frequency of the training programme varied between the studies. One study let patients train on their own and the frequency of the exercise during the training programme is not reported [33]. The remaining studies used counselling of a physiotherapist to train patients in allocated training sessions. Frequency of the programmes varies from one time per week, to five times per week. The total number of sessions patients had to participate ranged from 4 to 40 sessions.

### Outcome measurement

Five out of six studies included IRD as an outcome parameter. IRD was measured with a total of four different instruments (ultrasound, tape measure, palpation, and dial caliper). Three studies assessed IRD with two instruments simultaneously [31, 32, 34]. Overall, three studies used palpation as measurement for IRD, two studies combined palpation with tape measurement, or dial caliper measurement. One study used only tape measurement [33], and one used only palpation [30]. Two studies did not report if the IRD measurement took please during muscle contraction or relaxation. The remaining three studies reporting on IRD all measured IRD during muscle contraction. Four of the five studies measuring IRD reported the location of IRD measurement. One study measured at the umbilicus, two studies measured two centimetres above and below the umbilicus, and one measured four-and-a-half centimetres above and below the umbilicus.

### Results of physiotherapy training programme

Five out of six studies reported IRD as outcome measurement. Follow-up in all studies was performed directly after the training programmes ended. Hence, follow-up ranged from 2 weeks to 4 months. Five articles reported IRD as their primary outcome and all reported a decrease of IRD during the exercise programme. Both case reports describe IRD values below 22 mm during muscle contraction, after completion of the training programme.

The article of Emanuelsson et al. measured QoL using the SF-36 questionnaire and report that 87% (*n* = 26) of the patients were unsatisfied with the results of their training therapy and opted for surgical intervention after completion of the training programme.

## Discussion

Diastasis of the rectus abdominis muscles is a common problem. If not treated as part of an abdominoplasty in the field of plastic and reconstructive surgery, there is a lack of consensus on the preferred treatment of DRAM. Apart from conservative therapy, operative intervention and physiotherapy are the most frequently reported treatments for DRAM. This review provides an overview of the results of these interventions.

The overall quality of the included studies was low-to-moderate, combined with limited scientific power since only five prospective studies were included, of which two were RCTs. For this reason, meta-analysis of the included studies was deemed unfit. As stipulated by the review of Hickey et al. in 2011, indication for DRAM repair has different considerations compared to ventral hernia repair, despite the clinical similarity between the two entities [35]. Indication for DRAM repair is most often based on cosmetic or functional impairment, as DRAM poses no risk of strangulation. Therefore, the cosmetic results of a surgical technique or physiotherapy training programme, along with other patient reported outcomes (PROs), should be an important outcome of scientific studies. Remarkably, cosmetic outcome was only included in one study, and measured subjectively with an instrument that was not validated. Other PROs were not measured in surgical publications and only reported twice in physiotherapy studies.

### Surgical technique

Based on the published literature, the surgical techniques available for DRAM repair are either plication-based or hernia repair-based. The plication-based techniques include open plication, laparoscopic plication, or hybrid plication of either the anterior or posterior rectus fascia. Based on the results of this review, there is no clear difference in postoperative complications between these methods. Nearly all studies that described a plication technique used interrupted sutures and mesh reinforcement, which could account for the low recurrence rates, though comparative data are not available. The plication techniques can leave a surplus of skin directly after surgery, as described in the study of Sahoo et al., though due to the lack of cosmetic outcome measurement no evidence-based statements regarding the cosmetic postoperative appearance can be made [15].

Hernia repair techniques can be used for DRAM treatment. The musculofascial continuity of the ventral abdominal wall is an important anatomical structure to be considered during DRAM repair. If the midline is incised and the continuity is disturbed, the risk of incisional hernia formation and subsequent risk of strangulation will become larger, though alignment of the rectus muscles could be easier. The current evidence is of insufficient quality to detect differences between techniques that preserve the musculofascial continuity versus techniques that incise the midline. Hernia-based techniques for DRAM repair are often modifications of the original Chevrel or Rives–Stoppa techniques [21, 36, 37].

An important reason for DRAM patients to seek medical attention is the cosmetic impairment they experience. Despite the importance of cosmetic results, the majority (85% of patients) of published literature in general surgery for DRAM concerns open procedures. Recently developed hybrid techniques such as the ELAR plus described by Köckerling et al., the endoscopic midline plication by Bellido et al., or the eMILOS by Reinpold et al. could increase the number of minimally invasive procedures in the DRAM population [6, 26, 38]. These procedures are promising variations of classic (open) hernia repair techniques that respect the anatomical myofascial continuity of the ventral abdominal wall, and leave only minimal scarring, without risk of incisional hernia formation because the abdominal cavity is not opened. Given the recent invention of these techniques, the amount of published data is limited and with short follow-up. The study of Reinpold et al. unfortunately had to be excluded since the article did not describe critical data about the study methodology or the population [38]. Nevertheless, the technique seems to be promising for DRAM treatment.

It was not possible to isolate any difference in outcome between male and female patients of the included studies due to quality and reporting limitations of the included studies. It is the author’s opinion that the pathophysiology between males and females, or between type A/B and type C/D DRAM is different. The A/B type DRAM may be based on a physiological response during pregnancy, when collagen is remodelled under the influence of the hormone ‘relaxin’ to allow widening or strentching of the midline that is not corrected properly after pregnancy [39, 40]. In males, or type C/D DRAM, genetic predisposition or altered collagen 1:3 ratio’s may have a more pronounced role.

### Physiotherapy

The literature regarding physiotherapy interventions is heterogeneous in nature and of low quality. The type of exercises used to reduce IRD, the frequency of the exercises, the total number of sessions within a training programme, and the instruments used to asses IRD vary greatly amongst the included studies. For instance, the case study of Litos et al. informed their patient to avoid abdominal exercises that could increase IRD by recruitment of the transverse abdominal muscles, such as sit-ups, crunches, and rotational trunk exercises, whilst other studies from Ramesh et al. and Walton et al. target specifically the transverse abdominal muscles with these exercises to reduce IRD [31, 32, 34]. The included studies only report on postpartum women (type A, B), making translation of the results to men and type C and D DRAM difficult, if not impossible.

Brauman et al. has investigated the clinical anatomy of DRAM and reported that DRAM is not only associated with a gradual thinning and stretching of the linea alba, but also by a laxity of the ventral abdominal musculature [1]. Considering Brauman’s findings, physiotherapy could play a role in treating the laxity of the ventral abdominal musculature.

Despite the potential benefits of physiotherapy, current literature does not describe the successful treatment of rectus diastasis nor a reduction of IRD measured in a relaxed state. Since diastasis rectus is defined as a separation of the rectus abdominis muscles as measured in a relaxed state, we must conclude that the currently available evidence does not describe the successful treatment of rectus diastasis after a physiotherapy training programme. Physiotherapy was able to moderately reduce IRD during muscle contraction. The impact of these results on quality-of-life or functional outcome is currently unknown, as is the sustainability of these results after a follow-up exceeding four months. Based on the study of Emanuelsson et al., physiotherapy alone is unlikely to lead to satisfying functional and cosmetic results [5]. Emanuelsson et al. compared DRAM patients treated with a physiotherapy training programme with patients whom received abdominoplasty for DRAM in a randomised controlled trial. Eighty-one percent of the patients in the training group were unsatisfied with their functional and cosmetic appearance at the end of the programme and opted for surgical intervention after the trial ended.

Physiotherapy could be an alternative to surgery for patients who are unable or reluctant to undergo surgical intervention. Surgical treatment only corrects the widening of the linea alba and will not influence the general laxity of the ventral abdominal wall. Therefore, physiotherapy could be a useful addition to surgical intervention for DRAM, to achieve satisfying functional outcome.

### limitations

Most of the included studies were performed in postpartum women (rectus diastasis type A and B), reducing the translatability to men and rectus diastasis type C and D. Most of the included studies were retrospective and non-comparative in nature, reducing the scientific power of the review. The quality of the included studies according to the MINORS criteria was low; this is in part due to the ‘how-to-do-it’ type of publication which describes surgical techniques in scientific journals. These articles only include a small population, limiting the description of the randomisation, inclusion, end point, and blinding methods. Moreover, the MINORS score is sensitive for retrospective studies, as a retrospective study will automatically lose four out of sixteen points. Due to the low quality of the included studies, gender differences could not be isolated from the included population. Despite the above-mentioned limitations some general recommendations and conclusions can be drawn from this review.

## Considerations for DRAM treatment

### DRAM is not a hernia

The continuity of the myofascial anatomy in the ventral abdominal wall is what sets DRAM apart from an abdominal wall hernia. There are endoscopic, hybrid, and open techniques available that leave the anatomical myofascial continuity intact, and could potentially protect the DRAM patient from the risk of incisional hernia formation in case of a failed repair. Whether these techniques have any cosmetic or functional advantage over traditional hernia repair techniques, and if there is indeed no risk of incisional hernia formation, is currently unclear.

### Align and use mesh

For the repair of DRAM (without midline hernia), plication techniques with mesh reinforcement and interrupted sutures are most frequently used to reconstruct the ventral abdominal wall. Only using mesh reinforcement without an approximation of the rectus fascia of some sort, may lead to unsatisfying cosmetic results. Other minimally invasive, hybrid, or open techniques are promising and can be used, though long-term results and comparative controlled data are not available.

### Evaluate what is important for the patient

The current body of evidence focuses primarily on recurrence rates. It is well known that the risk of recurrence is not the most important variable for the patient. Instead, PROs such as postoperative pain, cosmetic outcome, and functional result are variables that directly concern the patients’ wellbeing. The use of PROs is low in both hernia- and DRAM-related research, and should be increased during the coming years [41].

### Cosmetic outcome is important

Cosmetic impairment is an important factor for DRAM patients to seek medical attention. Therefore, cosmetic result of a surgical intervention is of high importance in the DRAM population. Which surgical procedure (endoscopic, hybrid, laparoscopic, or open) has the most satisfying cosmetic outcome is not yet evaluated.

### Consider physiotherapy

Physiotherapy is unlikely to completely treat DRAM, since cases in which IRD was reduced to normal during a relaxed state are currently not described in literature. However, a moderate reduction in IRD during muscle contraction is reported. Whether this has an influence on functional outcomes or quality-of-life is not described. Physiotherapy combined with surgery could potentially have favourable results over surgery alone, this combination of treatments is currently not investigated. Moreover, patients that are reluctant or unable to undergo surgery may be referred to a physiotherapist for strengthening exercises of the abdominal wall.

### Recommendations for future studies

The authors would like to recommend future studies reporting on the efficacy of any DRAM repair to include PROs such as cosmetic outcome, quality-of-life, and work impairment, measured with a validated questionnaire (EuraHS QoL, COMI-Hernia) as their primary outcome, and IRD or recurrence rate as a secondary outcome. Both should be measured using an objective tool (dial caliper or ultrasound), as finger palpation should not be used for scientific outcome reporting [42]. The location of measurement and the cut-of value should be standardised for patients’ age, location of measurement, and pre- or postpartum status according to previously published classifications [2, 42–44]. IRD should be measured whilst the rectus abdominis muscles are relaxed, as references values of normal midline width and DRAM classification are measured/based on measurements during muscle relaxation. Based on the pathophysiology described by Brauman, the combination of physiotherapy and surgical repair has great theoretical potential to solve both the anatomical divarication and the laxity of the ventral abdominal muscles [1]. The authors recommend that future RCTs focus on the combination of surgery and physiotherapy for the repair of DRAM.

## Conclusion

Published literature on surgical treatments for rectus diastasis is of low scientific and methodological quality. Both plication-based methods and hernia repair methods are used for DRAM repair. Based on the current literature, no clear distinction in recurrence rate, postoperative complications, or patient reported outcomes can be made. DRAM is most frequently repaired using plication techniques combined with mesh reinforcement. Current literature does not describe the successful treatment of DRAM or a reduction of IRD in a relaxed state following physiotherapy. Physiotherapy can achieve a moderate reduction in IRD during muscle contraction, though it is currently unclear if this has any positive effect on quality-of-life or functional outcomes.
